# Stimuli-Responsive Hydrogels for Local Post-Surgical Drug Delivery

**DOI:** 10.3390/gels6020014

**Published:** 2020-05-08

**Authors:** Esfandyar Askari, Amir Seyfoori, Meitham Amereh, Sadaf Samimi Gharaie, Hanieh Sadat Ghazali, Zahra Sadat Ghazali, Bardia Khunjush, Mohsen Akbari

**Affiliations:** 1 Biomaterials and Tissue Engineering Department, Breast Cancer Research Center, Motamed Cancer Institute, ACECR, Tehran P.O. Box 1517964311, Iran; esfandyar.askari@gmail.com; 2 Laboratory for Innovations in Micro Engineering (LiME), Department of Mechanical Engineering, University of Victoria, Victoria, BC V8P 5C2, Canada; am.seyfoori@gmail.com (A.S.); m.amereh14@gmail.com (M.A.); s.f.sadaf@gmail.com (S.S.G.); bkhunjush@gmail.com (B.K.); 3 Nanotechnology Department, School of Advanced Technologies, Iran University of Science and Technology, Tehran P.O. Box 16846-13114, Iran; hani.qazali@gmail.com; 4 Biomedical Engineering Department, Amirkabir University of Technology (AUT), Tehran P.O. Box 158754413, Iran; Mahya.ghazali@yahoo.com; 5 Center for Biomedical Research, University of Victoria, Victoria, BC V8P 5C2, Canada; 6 Center for Advanced Materials and Related Technologies, University of Victoria, Victoria, BC V8P 5C2, Canada

**Keywords:** drug delivery systems, implantable, injectable, sprayable, hydrogel

## Abstract

Currently, surgical operations, followed by systemic drug delivery, are the prevailing treatment modality for most diseases, including cancers and trauma-based injuries. Although effective to some extent, the side effects of surgery include inflammation, pain, a lower rate of tissue regeneration, disease recurrence, and the non-specific toxicity of chemotherapies, which remain significant clinical challenges. The localized delivery of therapeutics has recently emerged as an alternative to systemic therapy, which not only allows the delivery of higher doses of therapeutic agents to the surgical site, but also enables overcoming post-surgical complications, such as infections, inflammations, and pain. Due to the limitations of the current drug delivery systems, and an increasing clinical need for disease-specific drug release systems, hydrogels have attracted considerable interest, due to their unique properties, including a high capacity for drug loading, as well as a sustained release profile. Hydrogels can be used as local drug performance carriers as a means for diminishing the side effects of current systemic drug delivery methods and are suitable for the majority of surgery-based injuries. This work summarizes recent advances in hydrogel-based drug delivery systems (DDSs), including formulations such as implantable, injectable, and sprayable hydrogels, with a particular emphasis on stimuli-responsive materials. Moreover, clinical applications and future opportunities for this type of post-surgery treatment are also highlighted.

## 1. Introduction

According to the National Institute of Health’s Global Health Research Unit, at least 4.2 million people worldwide die within 30 days of surgery annually [1]. Therefore, designing and developing efficient post-surgery treatments are crucial. The conventional therapeutic systems for post-surgery remission and treatment include utilizing systemic administration of therapeutic agents for preventing the recurrence of cancer, reducing inflammation in the injured tissue, and mitigating pain at the injury site [2,3,4]. In cancer, disregarding safe surgical margins during tumor resection surgery and the presence of cancer stem cells are two factors that might increase the risk of cancer recurrence. In particular, for cancer surgery, in order to reduce the risk of recurrence or metastasis, radiotherapy and systemic chemotherapy are used in patients with solid tumors. Nevertheless, some side effects, such as a lack of targetability and the incidence of drug-resistance over time, may arise during current systemic therapies [5,6]. In other diseases, vacant sites resulting from surgery are commonly filled with implants, primarily composed of polymeric or ceramic materials. These materials should have tissue regeneration capabilities, while simultaneously being degradable or capable of integrating within the host tissue. It should be also noted that these implants have the potential to generate inflammation and infection and reduce the rate of regeneration. [7].

The local delivery of drugs, nutrients, and other therapeutic agents used for cancer elimination, inflammation prevention, and the regeneration of injured tissue are promising tools for overcoming these challenges [8]. In localized therapy, including regional post-surgical cancer treatment, a high drug concentration is locally delivered at the specific tumor site. Accordingly, off-target drug toxicity is limited to within the tumor area, and the adjacent healthy tissues are not impacted [9,10]. Moreover, during district chemotherapy, the drug release rate is generally controlled. Accordingly, the probability of maintaining the drug level at the target site within the therapeutic window between the minimum effective concentration (MEC) and the minimum toxic concentration (MTC) increases [11]. In this regard, Qu and coworkers reported a biodegradable hybrid hydrogel comprised of gold nanorods (GNR) incorporated Methoxylpoly (ethylene glycol)-poly(ε-caprolactone)-acryloyl chloride (PECA) /glycidyl methacrylated chitooligosaccharide (COS-GMA)/N -isopropylacrylamide (NIPAm)/acrylamide (AAm) (PCNA) hydrogel (PCNA-GNR hydrogel) for the post-surgery treatment of breast cancer. Doxorubicin, a chemotherapeutic agent, was loaded into the hybrid hydrogel to inhibit breast cancer recurrence. These hybrid hydrogels degrade 14 days after implantation [12]. Localized therapy after surgery is mostly conducted by using drug delivery depots or carriers for the sustained or on-demand release of drugs. These carriers are primarily made of soft polymers, and can be applied in different physical forms such as gels, wafers, films, or microparticles [13,14,15]. The major advantage of these systems is the biodegradability of the drug delivery carriers, which eliminates removal surgery and avoids a chronic foreign-body immune response [16].

Among the current depot delivery systems for localized therapy, hydrogels have attracted great attention. Generally, hydrogels benefit from specific properties, including elastic networks, which provide a large swelling capacity with the subsequent high levels of drug loading ability in their structure. Therefore, hydrogels are considered powerful platforms, through which the requirements of local drug delivery to different diseased tissues can be fulfilled. Based on chemical composition and physical shape, hydrogels can be used in a variety of other bio-engineering applications, including protein enrichment [17], cell isolation [18,19], and high-density cell expansion [20]. According to the literature and the commercial market, bio-engineered hydrogels have been applied in three main forms: implantable hydrogels, injectable hydrogels, and sprayable hydrogels. In this regard, the aim of this article is to review the most recent hydrogels utilized for localized post-surgical drug delivery and their clinical usage in wound healing, cancer treatment, spinal, and periodontal surgeries. In addition, the benefits of various smart and stimuli-responsive hydrogels for on-demand drug release are elaborated upon.

## 2. Implantable Hydrogels

Implantable hydrogels are 3D stable hydrogels, in which the polymeric chains (natural or synthetic) of hydrogels are cross-linked by chemical and physical interactions [21]. In chemically cross-linked hydrogels, covalent bonds generally result in a stronger and more stable network, and chemical degradation or other strategies are then essential for the removal of the hydrogels from a biological environment [22]. Different polymerization reactions can occur during the covalent cross-linking process. The conventional reactions include click chemistry, thiol-end chemistry, and free radical polymerization, which generate mechanically enhanced hydrogels. Mechanisms and details of these reactions have been previously reported by Hu et al. [23]. Distributed nanoparticles within hydrogels have been applied as a reinforcement agent to enhance the hydrogel’s mechanical properties. Spherical, cubic, and sheet forms of nanoparticles can interact with hydrogels’ polymeric chains via hydrophobic attraction, electrostatic interactions, or covalent-based interactions [24]. Such interactions enhance the mechanical properties of the matrix and enable hydrogel implantation at different surgical sites [25].

**Figure 1:**
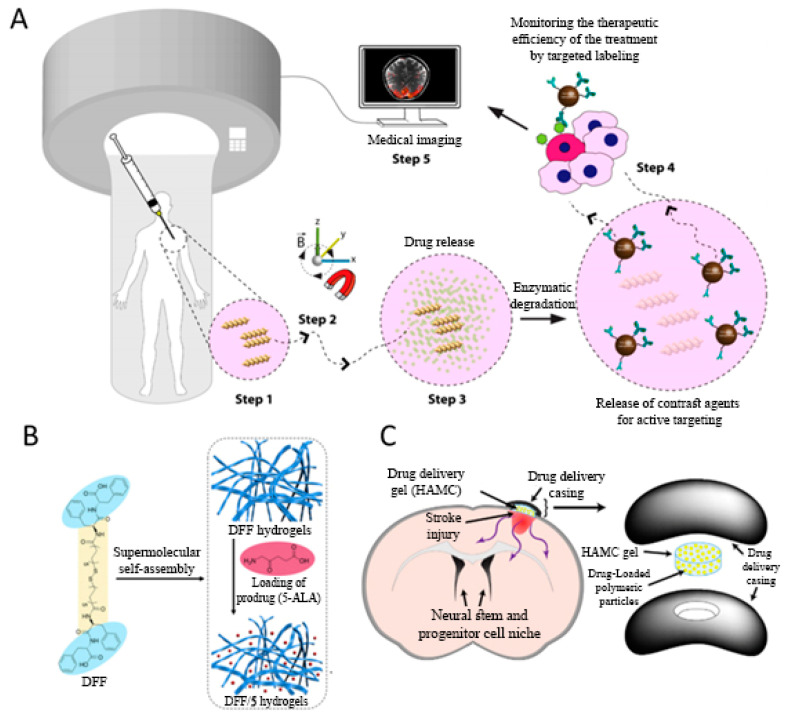
(**A**) Schematic view of the application of the 3D-printed micro-robotic swimmers. Micro-swimmers are directed to the disease or intervention site using an external magnetic force. Tumor microenvironment pathological condition led to the enzymatic degradation of the micro-swimmer by matrix metalloproteinase-2 (MMP-2), and boosted labeled-magnetic nanoparticle release. Antibody-modified magnetic contrast agents diffuse around to label the untreated tissue sites, Reprinted with permission from American chemical society. Adapted with permission from Ceylan et al. [33]. (**B**) Illustration of the composition of fibrous bola-dipeptide hydrogels for localized and sustained delivery of prodrug towards the tumor site. Adapted with permission from Zou et al. [45]. (**C**) Poly (lactic-co-glycolic acid) (PLGA) microparticle loaded hyaluronan/methylcellulose (HAMC) hydrogel for local drug release to the stroke injured brain tissue. The exact location of the hydrogel is held in place by both gelation and a casing comprised of polycarbonate discs. Adapted with permission from Tuladhar et al. [49].

Three-dimensional (3D) printing is a promising technique for fabricating implantable hydrogels for various biomedical applications [24,26,27,28,29]. These types of hydrogels have been reported in the literature as a potential scaffold for post-surgery applications in in vivo experiments [30,31]. However, there are just few numbers of these printable scaffolds, like poly-lactic acid (PLA) hydrogels, which are approved by FDA. In addition, photo cross-linkable hydrogels have been widely used to fill the vacant site after surgery [32]. According to the photo-curability of cytocompatible hydrogels, ultraviolet (UV)-assisted 3D printing has been introduced to fabricate hydrogels with defined features, such as complex geometry and excellent mechanical properties, to effectively fill the surgical site. Ceylan and coworkers fabricated a 3D printed biodegradable microswimmer for theranostic cargo delivery and release [33]. Micro robotic swimmers were fabricated from gelatin methacryloyl (GelMA), known as a cytocompatible and photo-crosslinkable hydrogel. Magnetic nanoparticles (MNP) were conjugated with the ErbB2 antibody and added to the GelMA solution. Printed micro-robotic swimmers were degraded in the enzymatic environment, and released the MNPs for targeted cell labeling of ErbB2 overexpressing SKBR3 cancer cells (Figure 1A) [33]. The unexpected interaction of drugs, nutrients and other agents with the photopolymers is the main challenge of photocurable 3D printed fabricated delivery systems, as was first reported by Xu et al. They illustrated that the diacrylate group of the photoreactive monomer and the primary amine group of amlodipine formed a Michael addition reaction [33].

In contrast to chemically cross-linked hydrogels, physical hydrogels are formed by non-covalent interactions, including hydrogen bonding, ionic interactions, and hydrophobic interactions. Self-assembly, crystallization, and thermally induced crosslinking are the main cooperative interactions used to generate physical hydrogels. The major advantage of physically cross-linked hydrogels to chemically cross-linked ones is the feasibility of hydrogel formation in the presence of physical environmental cues. In this regard, the risk of any toxicity incident for the target tissue is avoided. Nevertheless, the stability of physical hydrogels can be altered by changes in pH, temperature, and non/aqueous solvents. For example, peptide chains form a self-assembled hydrogel via hydrophobic interactions, without the use of any cross-linker and initiator, which eliminates any potential cytotoxicity and side effects. Despite the green and simple formation process of physical hydrogels, their weak mechanical properties are still considered as a challenge [34].

The aforementioned strategy for the formation of physical hydrogels is discussed below:

### 2.1. Crystallization-Induced Cross-Linking

A crystalized hydrogel is generally called to subgroup of hydrogels, in which polymer segments generate a high structural symmetry and tacticity [35]. Given the presence of an ordered structure of polymer chains in the crystalline phase, compared to the amorphous phase, using this feature is effective to enhance the strength of cross-linking in the hydrophobic–induced physical hydrogels. Poly (vinyl alcohol) (PVA) as an FDA approved material, is one of the most widely used hydrogels, which are cross-linked through crystallization. The freeze-thawing process is one of the methods used to form crystallized polymers. In this process, freezing the water within the polymer chains forms regions of very high polymer concentration, drawing the polymer chains together and facilitating their cross-linking [36]. The swelling and mechanical properties of crystallization induced physical hydrogels are mainly controlled by the freeze-thaw cycle. Holloway et al. show that repeated freeze-thawing may significantly enhance the mechanical properties of PVA physical hydrogels by inducing the formation of secondary crystallites, and providing the hydrogels with stability at room temperature [36].

Controlling the mechanical properties of the fabricated PVA 3D hydrogels for post-surgery treatments is a considerable factor. In this regard, Zhang et al. illustrated that synergistic combinations of different crosslinking mechanisms in an interpenetrating polymer network (IPN) structure enhance the mechanical properties of PVA hydrogels [37]. Ooi and coworkers also produced a cellulose nanocrystal reinforced gelatin hydrogel by increasing the overall crystallinity of gelatin hydrogel, which, in turn, improved the hydrogel’s mechanical properties [38].

### 2.2. Self-Assembly-Induced Cross-Linking

A large number of biomacromolecules, including peptides and proteins, can form network structures by forming coiled-coil, triple helix, and β-sheet structures. Collagen-based and silk-based hydrogels are some examples of self-assembling hydrogels. L-Phe-L-Phe (FF), a derivative of amyloid fibrils, is one of the popular peptides building blocks for self-assembly. Various types of nanostructures based on FF and its derivatives have been introduced in different studies. It has been reported that FF by itself cannot form hydrogels [39,40]. Synthetic conjugates of FF and aromatic groups or longer peptides containing the FF motif are reported as hydrogel forms of FF [41,42]. These hydrogels have been shown to have a structure that has the potential for biomedical applications. One of the main challenges in FF modification is the reduced biocompatibility of the resulting hydrogels (at high concentrations of modifier). A Fluorenylmethyloxycarbonyl (Fmoc) group is widely utilized to modify the FF structure and promote the hydrogen forming ability of FF. Nevertheless, there are some limitations, and Fmoc-linked FFs lose their stability in physiological mediums [43]. Bola-type amphiphiles are recognized as self-assembling building blocks, derived from the specific architecture of monolayer membranes in archaebacteria [44]. The major mechanism in the formation of bola-type self-assembled hydrogels is the hydrophobic interaction of the intervening structures located between the two hydrophilic groups. Zou et al. presented a self-assembly strategy based on bola-dipeptides for designing injectable peptide hydrogels for localized and sustained drug delivery [45]. This strategy involves the synthesis of a novel designed bola-dipeptide (a di-FF derivative, DFF) and the formation of fibrous bola-dipeptide hydrogels in an aqueous solution (Figure 1B). The resulting hydrogels have the advantages of good stability, rapid recovery, and excellent biocompatibility, without the need for synthetic aromatic groups. Glyoxylamide-based short peptides is a new group of hydrogels which have the ability to self-assemble and form stable hydrogels. These groups are obtained via a ring-opening reaction of N-acylisatin. Glyoxylamide-based hydrogels show long-term self-assembling properties, and have a sustained release rate of antibacterial agents, which makes this hydrogel a suitable candidate for antibiotic delivery applications [45].

Anthranilamide is structurally similar to glyoxylamide, and can be synthesized by a ring-opening reaction of isatoic anhydride, with the use of primary amine acting as a nucleophile. Anthranilamide possesses an aromatic group which can generate π-π interactions, and hydrogen bonding sources that can participate in intermolecular interactions. Anthranilamide-based short peptides formed a self-assembled hydrogel, which was triggered by changes in pH, heat, and solvent. Applying substituents on the capping group and changing the hydrophobicity are the parameters that have significant effects on Anthranilamide based hydrogels [46].

### 2.3. Thermal Cross-Linking

The term thermal cross-linking is dedicated to types of polymers that, at around a specific temperature—named the low/high critical solution temperature—alter their physical characteristics and undergo a transition from a hydrophilic to hydrophobic state, or vice versa. Through this transition, physical cross-linking and structural changes from solution to gel occurrs [47]. Poly N-isopropylacrylamide (PNIPAAm) is known as a thermo-responsive FDA-approved polymer, which can be used in the self-assembly and formation of materials for biomedical and drug delivery applications [48]. Hyaluronan and methylcellulose are other cytocpmpatible polymers which can form physical hydrogels using the thermal gelatin process. The potential of this hydrogel for local delivery has been evaluated by Tuladhar et al. [49]. In the reported system, the hydrogel was used as a matrix for poly (lactic-co-glycolic acid) (PLGA) microparticles loaded with different drugs, as shown in Figure 1C. It was demonstrated that the sustained release of cyclosporin A (CsA) from the PLGA microparticles over 14 days resulted in more significant effect, in comparison with systemic therapy. Furthermore, this method produced a higher local CsA concentration in the brain tissue, and reduced drug exposure in other organs.

## 3. Injectable Hydrogel

**Figure 2:**
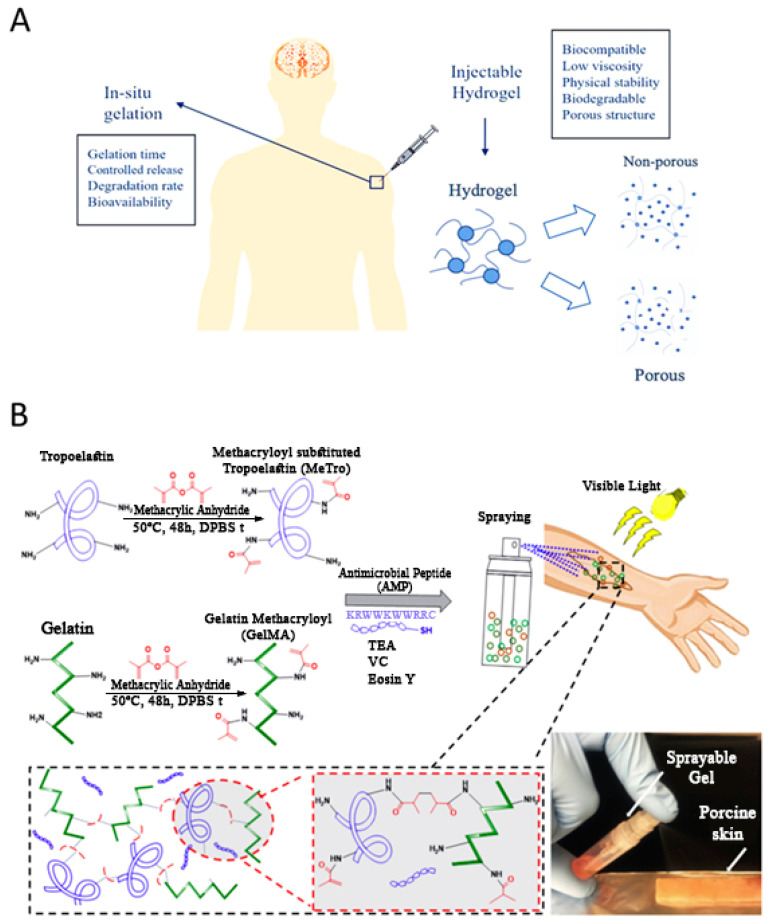
(**A**) Schematic illustration of injectable hydrogels in localized therapeutic agents’ delivery. It shows the required physical characteristics of the primary sol and as-injected gel for an ideal localized drug delivery depot. (**B**) Design and chemical composition of methacryloyl-substituted recombinant human tropoelastin (MeTro)/gelatin methacryloyl (GelMA)-AMP composite hydrogels. Schematic reaction of MeTro, GelMA, and AMP, after adding to TEA (co-initiator) and vinyl caprolactam (VC) (co-monomer) solutions. Eosin Y (photoinitiator) was finally introduced into the solution to become ready for spraying onto the wound area and exposing to visible light. Adapted with permission from Annabi et al. [69].

The lack of drug permeability in deeply located tissue injury sites has made hydrogels an attractive option for the treatment of said wounds. As mentioned above, implantable hydrogels were initially studied for this application, but the potential risks and inconveniences of surgery altered research trends towards injectable hydrogels as a possible solution [29,50]. Injectable hydrogels are a good candidate for creating a 3D microenvironment in the human body. As shown in Figure 2A, they need to possess several specific features in order to be biocompatible and biodegradable. The injectable hydrogels should have low viscosity prior to gelation, as they need to present a microenvironment that allows cells or drugs to homogeneously disperse while keeping their bio-availability and function. Additionally, the pore size and the interconnectivity of the pores must provide a suitable space for cellular activity, and for the exchange of oxygen and other nutrients essential for cell growth. Moreover, the gelation rate after injection of the biomaterial should be suitable for proper drug encapsulation, while avoiding toxicity, boosting drug release, and impacting the adjacent healthy tissues [51]. Generally, using injectable hydrogels, which provide local drug delivery in cancer chemotherapy and other disease treatment, has many advantages over implantable hydrogels. These advantages include the selective and sustained release of drugs to the diseased tissue in a minimally invasive manner. Depending on the type of matrix material, injectable hydrogels are classified into two groups of natural and synthetic hydrogels. However, there are many alternatives to make multi-component injectable hydrogels by combining the natural and synthetic compartments. Herein, there is great opportunity to easily modify the physical and rheological properties of them, by making combined hydrogels, or even nanocomposite hydrogels incorporated with nanostructures inorganic/organic materials.

### 3.1. Natural Injectable Hydrogels

Chitosan is a cytocompatible polysaccharide derived from chitin deacetylation with biodegradable properties. Thermosensitive Chitosan hydrogels can be used in surgical implantation with in situ gelation [52]. Qu et al. suggested a series of pH-sensitive chitosan-based hydrogels as a drug carrier for Hepatocellular carcinoma (HCC), as it is one of the most common lethal cancers [53]. For preparing the hydrogel, a Michael reaction was used to synthesize N-carboxyethyl chitosan (CEC) in an aqueous solution, and in the presence of dibenzaldehyde-terminated poly (ethylene glycol) (PEGDA). They also encapsulated Doxorubicin, an anti-cancer drug, into the hydrogel structure in situ. Altering the concentration of the cross-linker has been used to adjust the hydrogel’s properties, such as the gelation time and gel degradation rate, through which the DOX release rate is controlled using different pHs. CEC/PEGDA rapidly formed a hydrogel in vivo after subcutaneous injection with minimal diffusion in the surrounding tissue. Moreover, drug release from the CEC/PEGDA hydrogel was efficient in causing cancer cell death and tumor burden shrinkage in the animal model [53].

Hyaluronic acid (HA) is the only non-sulfated glycosaminoglycan that can be found in connective, epithelial, and neural tissues. It is a natural polymer, which shows neither an immunogenic nor an inflammatory response. HA is one of the main components of the extracellular matrix (ECM) of most epithelial-based tissues, and exhibits a significant role in wound healing, angiogenesis, and also contributes to cell migration and proliferation [51]. Due to its bio-functionality, and having various modifiable sites for substituting reactive groups, HA is a good candidate as a drug carrier [54]. Highley et al. designed a system with adamantane-modified and β-cyclodextrin (CD)-modified hyaluronic acid as its main constituents, in order to create an injectable hydrogel capable of sustained releasing small molecules [54]. Conjugating Cyclodextrin to HA increases HA’s affinity for small hydrophobic molecules, such as tryptophan. Since cyclic domains, like the indole moiety in tryptophan, are found in many pharmacologic small molecules, the increased affinity of HA for tryptophan would facilitate the use of many hydrophobic small drug molecules for encapsulation in HA as a drug carrier [55].

Alginate, a linear polysaccharide extracted from seaweed, is also a promising candidate for use as an injectable hydrogel, due to its biocompatibility, controlled degradation features, and its simple crosslinking process with divalent cations [56]. Yang et al. designed an injectable and biodegradable gel scaffold based on alginate with hydroxyapatite (HAp) and gelatin microspheres (GMs) crosslinking by the in situ releasing of calcium cations [57]. In order to increase the bioactivity of the hydrogel scaffold, Tetracycline Hydrochloride (TH) was encapsulated in the GMs, and was released into the surrounding tissue in a controlled manner followed by injection. By adding HAp and GMs to the hydrogel structure, the bioactivity and the mechanical characteristics of the composite hydrogel were enhanced. The prepared composite hydrogel showed the suitable adjustable weight loss, swelling ratio, and gelation period, that is mandatory for use as a scaffold in bone tissue engineering [57]. Similar to the other example regarding cancer therapy, Liu et al. designed an injectable thermoresponsive hydrogel using alginate-g-PNIPAAm loaded with doxorubicin (DOX) [56]. The steady and slow release of DOX, by the injectable alginate-based hydrogel, improved the killing rate of the highly drug-resistant cancer cells [56].

Collagen is the most abundant protein in mammalian tissues, and is an essential part of the ECM, which makes it very useful in tissue engineering applications. However, self-assembled collagen hydrogels have noncovalent interactions, such as hydrogen bonds, resulting in poor mechanical properties, and have limited applications as a drug carrier system. The required mechanical properties of the hydrogel can be improved by adding nanoparticles to the hydrogel’s network. Xing et al. synthesized collagen-based hydrogels containing gold nanoparticles with adjustable mechanical properties [58]. This hydrogel can be used for locally maintaining drug release to decrease toxicity in the microenvironment and reduce the required dosage [58].

Gelatin is another protein-derived hydrogel consisting of single-strand molecules, and is obtained from denaturing the origin triple-helix structure in collagen. Gelatin is biocompatible, biodegradable, and readily available, which makes it an excellent choice for biomedical applications [51]. Wang et al. prepared an injectable composite hydrogel comprised of gelatin (GE)/oxidized alginate (OSA)/adipic acid dihydrazide (ADH) with self-healing properties and a macro-porous structure [59]. The GE/OSA/ADH hydrogel provided a novel and safe drug carrier for biomedical applications. The structure maintains a liquid state for some minutes at room temperature, but upon injection, it quickly gelates in vivo [59].

Chondroitin sulfate (CS) serves as another injectable hydrogel with covalent or non-covalent functional groups, which can be utilized for adjusting its biodegradation characteristics. By using anionic groups in the structure of the hydrogel, electrostatic interactions facilitate the absorption of drugs with cationic groups into the hydrogel’s structure, obtaining drug-eluting hydrogels as a localized injectable carrier. As an example, Ornell et al. introduced an adjustable CS modified with methacryloyl groups (CSMA), blended with PVAMA [60]. The CSMA/PVAMA hydrogels provide a sustained release of drugs during the six-week study, and have the potential for use in oncology therapeutic [60]. For this use, polysaccharides as gelling agents are the other groups of hydrogels that have attracted attention due to their biocompatibility, bioavailability, and bioactive functionality. In this regard, Jang et al. studied the injectable properties of Pinus Koraiensis Polysaccharide (PKP) for use as an injectable drug carrier hydrogel in cancer chemotherapy, to reduce the side effects of paclitaxel (PTX). PTX-loaded PKP greatly suppressed tumor growth in vivo and decreased toxicity [61].

### 3.2. Synthetic Injectable Hydrogels

Polyethylene glycol–poly lactic acid-co-glycolic acid (PEG–PLGA) is a synthetic copolymer with a tunable biodegradation rate and good mechanical properties [51]. For angiogenesis in scaffolds with interconnected pores, modified PEG–PLGA hydrogels can deliver active heparin and growth factors if they are used as a matrix. In fact, these gels have the potential for various tissue engineering applications that require angiogenesis, such as in growth matrices in vascular grafts or injectable gels for myocardial infarction therapy [62]. Posadowska et al. developed an injectable system for intra-bone delivery of Alendronate loaded nanoparticles (NPs-Aln) within a hydrogel matrix (gellan gum, GG), to eliminate limitations in phosphonates, such as low bioavailability and high toxicity [63]. Aln is also encapsulated in the poly (lactide-co-glycolide) (PLGA 85:15) by using the oil/water (o/w) emulsion method. All of the encapsulated drugs were released within 25 days, with the release ratio decreasing over time. However, NPs-Aln suspensions in the GG-based matrix had a significantly lower and steadier release rate. The engineered GG–NPs-Aln hydrogel could be injected without difficulty and maintained its structure during extrusion, which was established by rheological experiments. In vitro assessments with osteoblast-like cells proved the cytocompatibility of the hydrogel and its inhibition of osteoclasts activity. Moreover, the above-mentioned system enabled local sustaining of small amounts of Aln while enhancing biological activity, which makes the system a promising candidate for bone tissue engineering [63]. Yu et al. developed an injectable silk fibroin-polyethylene glycol (Silk-PEG) hydrogel as a carrier of poorly soluble micronized dexamethasone (m-DEX), which is an important and non-invasive medical treatment of inner ear disease [64]. The results showed that the gelation time of Silk/PEG hydrogel is greatly affected by the amount of loaded m-DEX [64]. PLGA–PEG–PLGA (PPP) is also one of the triblock copolymers that is widely used due to its biocompatibility, non-toxicity, and biodegradability. This block copolymer is not approved by FDA, but consists of the FDA approved segments PLGA and PEG individually. This copolymer is widely used as an injectable gel, as it can be injected at low temperatures and is easily converted into a gel in vivo, without the need for surgery [65]. Another synthetic polymer is polycaprolactone (PCL). In PEG-containing hydrogels, Poly(e-caprolactone) (PCL) blocks can be used instead of PLA and PLGA, which have a lower degradation rate and ultimately don’t produce an acidic environment [51]. Deng et al. formed a hydrogel from PEG hydrophilic blocks and PCL hydrophobic blocks for targeted and sustained drug delivery [66]. They evaluated the effect of the PEG / PCL ratio and molecular weight on gelation temperature, gel properties, and drug release kinetics. In cancer therapy and tumor cell killing, a pair of hydrogels based on PEG and polyesters have been suggested as a promising candidate for drug delivery vehicles [66]. As another example, recently shear-thinning injectable hydrogel composed of gelatin and laponite was developed for localized drug delivery applications [67]. It was shown that adding chitosan and PNIPAM microgels in the structure of the hydrogel made it pH responsive, while its shear thinning properties were preserved.

Table 1 summarize the whole injectable hydrogel compositions and their relevant applications. Some of these hydrogel systems are FDA approved or at least contain one FDA approved component.

## 4. Sprayable Hydrogels

**Figure 3:**
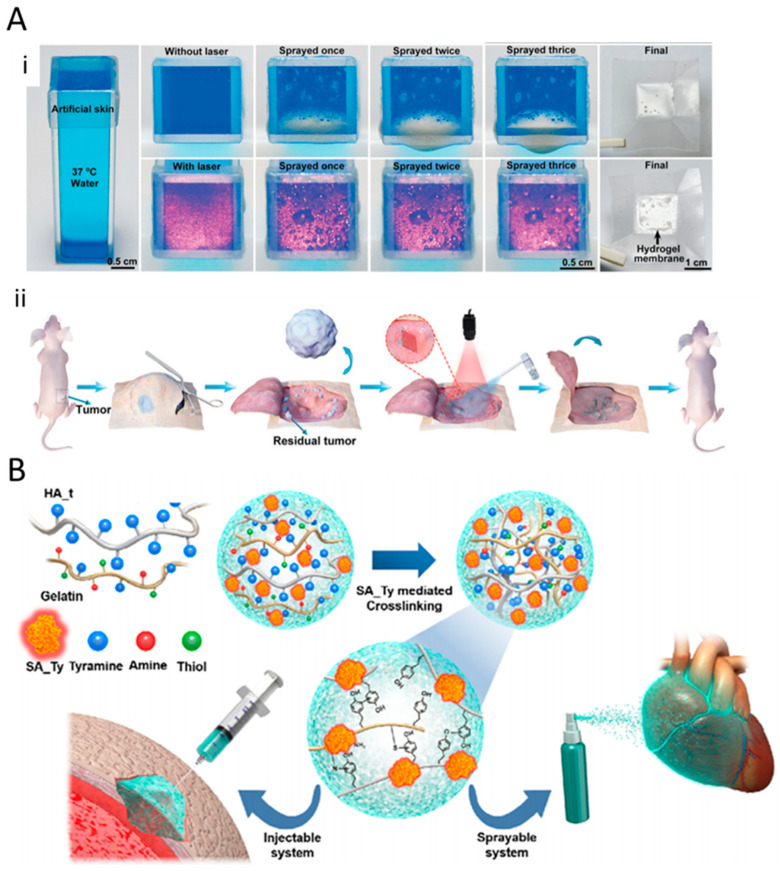
(**A**) (i) Camera photographs of BP@PLEL hydrogel sprayed on a piece of 37 °C artificial skin with (down) and without (up) 808 nm laser irradiation. Hydrogel membrane is formed in response to the near-infrared (NIR) laser. (ii) Schematic of the surgical treatment and postoperative photo-thermal therapy of the tumor at the site of the sprayed hydrogel. Adapted with permission from Shao et al. [71] (**B**) Illustration of the fabrication of sprayable system comprising hyaluronic acid (HA) hydrogel, crosslinked with SA-Ty mediated macromolecules (gelatin, HA_t), coupled with a neighboring amine, thiol, and another quinone. Adapted with permission from Kim et al. [72].

Another type of hydrogels, which has been mentioned as a potential candidate for use in post-surgery treatments, are sprayable hydrogels. In this type of hydrogel, polymer chains are cross-linked in situ (surgery site, damaged skin, and damaged tissue). These types of hydrogels are known for their user-friendliness. Annabi et al. introduced a visible-light cross-linkable sprayable-hydrogel, which consisted of gelatin methacryloyl (GelMA) and methacryloyl-substituted recombinant human tropoelastin (MeTro) [69]. Both GelMA and MeTro are derived from native ECM proteins. UV-based photo cross-linking processes, which have been applied to polymerize various types of hydrogels (see implantable section), induce DNA and tissue damage. Annabi et al. eliminated this concern by replacing the UV light with visible light [69]. Synergistic association of the two biopolymers enhanced the mechanical properties and modified the in vitro and in vivo degradation rate of the hydrogel system. GelMA-MeTro sprayable hydrogels show anti-microbial properties, which earmarked them as a promising adhesive gel for use in wound healing (Figure 2B) [69]. In the other study, Yoon et al. developed sprayable gelatinhydroxyphenyl propionic acid (GH) hydrogels, which can deliver MSC-attracting chemotactic cytokines to induce enhanced diabetic wound healing [70]. They reported that the enzymatic cross-linking reaction is the major mechanism of hydrogel formation for GH [70]. In the cancer treatment, Shao et al. reported a sprayable photo-thermal therapy (PTT) system comprised of black phosphorus (BP) nanosheets and thermosensitive hydrogel Poly(d,l-lactide)-poly(ethylene glycol)-poly(d,l-lactide) (PDLLA-PEG-PDLLA: PLEL) for the post-surgery treatment of cancer (Figure 3A) [71]. It was shown that, under 808 nm laser irradiation, the sprayed hydrogel forms a gel on the wound. This hydrogel offers a high photothermal efficacy for eradicating the remaining tumor cells after resection surgery, inhibiting tumor recurrence.

As another example, Kim et al. reported a sprayable tissue adhesive and rapid forming ECM hydrogel, using recombinant tyrosinase protein as the crosslinking agent [72]. Second-to-minute crosslinking kinetics are observed in the tyrosinase-based crosslinking process. In this study, the phenolic groups of hyaluronic acid (HA) are instantly oxidized by the Streptomyces avermitilis-derived tyrosinase protein, which resulted in the rapid crosslinking of the HA structure. They illustrated that the recombinant enzyme-based crosslinking method, not only enhanced the mechanical properties of the HA hydrogel, but also decreased the crosslinking time from minutes to seconds, which makes this hydrogel well suited for use as a sprayable hydrogel (Figure 3B) [72].

Fibrin gel is a US Food and Drug Administration approved material, and is formed by the interaction of fibrinogen and thrombin. Chen et al. reported a smart sprayable fibrin hydrogel comprised of fibrinogen, thrombin, and antiCD47 antibody loaded CaCO3 nanoparticles (aCD47@CaCO3) as an immunotherapeutic gel for preventing cancer recurrence after surgery [73]. pH-sensitive nanoparticles are embedded in the in situ formed fibrin hydrogels. Changes in pH at the sprayed site trigger the release of immunotherapeutic agents and prevent cancer recurrence [73].

Apart from the classification of the smart hydrogels based on their physical shape, they can be divided in to two subgroups, based on their physicochemical properties, called exogenously and endogenously triggered hydrogels. Exogenously triggering dedicated to hydrogels, which can respond to external stimuli like temperature, light or ultrasound, while endogenously triggering belongs to types of hydrogels which respond to specific stimuli within the body microenvironment including oxidation-redox reactions, pH changes, inflammatory, and enzymatic reactions. Depending on their chemical structure, hydrogels can respond to single or multi-stimulus in tissue microenvironment, so that by modifying their polymer structure, multifunctional smart hydrogels with the ability to offer multi-drug delivery with an on-demand release profile are developed.

## 5. Exogenously Triggered Drug Release

### 5.1. Thermo Responsive Drug Release

**Figure 4:**
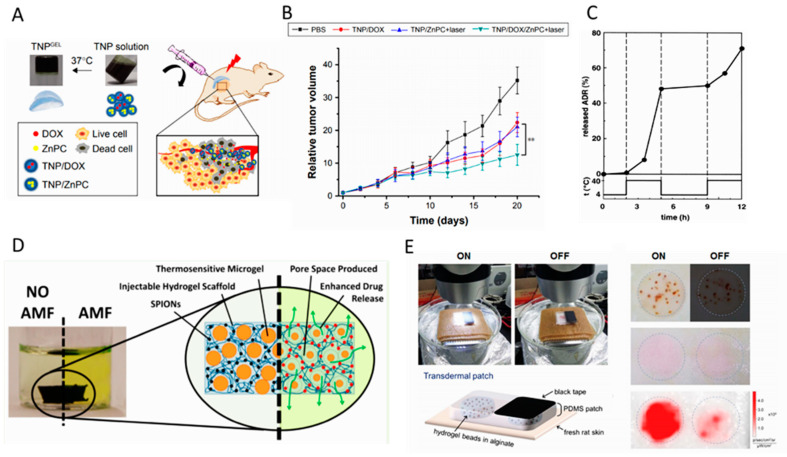
(**A**) Illustration of photo/chemo combination therapy via in situ formed thermal-sensitive polymer hydrogel (TNP) in a xenograft tumor model. Doxorubicin and zinc phthalocyanine loaded TNP polymer solution is transformed to gel state after injecting, to be used for localized photodynamic therapy. Adapted with permission from Huang et al. [77]; (**B**) relative tumor volume of the mice model in different treatment conditions showing the significance of concurrent localized chemo/PDT therapy. Adapted with permission from Huang et al. [77]. (**C**) Thermal fluctuations and corresponding on/off switch release of drug from thermo-responsive PIPAAm-PBMA micelles through the low critical solution temperature (LCST). Adapted with permission from Chung et al. [82]. (**D**) Hydrazide-functionalized poly N-isopropylacrylamide (PNIPAAm) microgels in a magnetic nanoparticle-containing matrix using for pulsatile on/off release of drugs in response to an external magnetic field. Adapted with permission from Campbell et al. [83]. (**E**) Ex vivo setup of the light-triggered release of the skin patch with drug reservoirs containing rhodamine-loaded hydrogel beads. It is shown that by the exposure of the visible light (LED lamp (30 mW·cm ^−2^) for 6 h), rhodamine is released from the Polydimethylsiloxane (PDMS)skin patch, which is significantly higher than that of “off “condition. Adapted with permission from Kim et al. [89].

Due to their huge potential in biomedical applications, thermo-responsive hydrogels have attracted great attention in the past decade. These hydrogels change their swelling properties in response to temperature gradients, and subsequently deliver various therapeutic agents. Indeed, temperature change serves as the signal for drug release modulation [74,75]. According to the above-categorized bioresponsive hydrogels, thermo-responsive hydrogels can be applied in either the implantable or injectable form. Thermo-gelation is the main synthesis mechanism of injectable thermo-responsive hydrogels, through which microgels participate in an in situ gel forming, above their low critical solution temperature (LCST) [29]. As an example, a poly(N-isopropylacrylamide)-chitosan (PNIPAAm-CS) sol-gel system was used for sustaining the delivery of timolol maleate as an intraocular pressure (IOP) reducing drug. By using this thermo-gelling hydrogel bio-availability, the efficacy of the timolol maleate was greater than in the conventional eye drops [76]. The other in situ thermo-gelation system was achieved through the co-polymerization of 3-caprolactone,1,4,8-trioxa [4.6] spiro-9-undecanone and poly (ethylene glycol), through which chemotherapeutic drug-loaded nanoparticles were assembled and formed a hydrogel at 37 °C (Figure 4A). As depicted in Figure 4B, this hydrogel was an effective drug carrier for the ablation of bladder tumors in mice models so that relatively high levels of reactive oxygen species (ROS) (a 4.8-fold increase) were generated, in comparison to free drug [77].

In another study, a microspheres-based thermo-responsive hydrogel was prepared on N-isopropylacrylamide using the emulsion polymerization method [78]. The size of the microspheres was found to be a key parameter for significantly improving the control of drug release. However, the model was not able to accurately respond to cycling temperature changes. Considering the utilization of thermo-sensitive hydrogels for on-demand drug delivery application, G. Cirillo et al. studied the releasing behavior of hydrogel films synthesized using UV-initiated radical polymerization, as a delivery method for anti-inflammatory drugs [79]. They investigated the hydrogel dependency of swelling/deswelling, modulation of diffusional constraints, and the release profile of the hydrogel. A higher release rate above the LCST at 40 °C was reported, as a result of hydrophobic interactions in NIPAAm moieties. Co-polymers of NIPAAm with three different alkyl methacrylates (RMA) monomers, (butyl methacrylate) BMA, hexyl methacrylate (HMA), and lauryl methacrylate (LMA), were studied as thermo-sensitive hydrogels for controlling drug release patterns [80]. Stepwise temperature changes were tested on these hydrogels, and the results showed that the capacity for accurate regulation in indomethacin release depended on the alkyl side-chain length.

As the next example of a developed thermo-responsive drug delivery system (DDS), 5-fluorouracil (5-FU) was loaded into PNIPAAm hydrogel [81]. In order to analyze the drug release pattern, a UV spectrophotometer was utilized to screen the release. It showed that the controllability of the release rate, which was faster at higher temperatures than at lower temperatures, was improved by using PNIPAAm hydrogel. As reported in the paper, this control release mechanism has a promising potential for DDs. Poly(N-isopropylacrylamide) PNIPAAm gels have also been used as an on demand (on–off module) DDS. For instance, thermo-responsive polymeric micelles were utilized to achieve both a passive and an on-demand stimuli-responsive function [82]. The model allowed drug release controlled by thermal fluctuations, and with a corresponding on/off switch through the LCST, due to its reversible structural properties, as shown in Figure 4C. The results of this study showed that the high sensitivity and reversibility of the micelle’s thermal response, in conjunction with localized hyperthermia, could be used as a successful model in DDS. As the other example of the pulsatile (on–off) thermoresponsive drug release, Campbel et al. reported a thermo-gelling hydrogel, including thermoresponsive hydrazide-functionalized PNIPAAm microgels, as well as magnetic nanoparticles incorporated in the matrix of an injectable hydrogel. This injectable system was used for the non-invasive on-demand release of drugs in response to exogenous stimuli, such as an alternative magnetic field (Figure 4D) [83]. A PNIPAAm-derived hydrogel was also used to fabricate dermal patches to generate a controlled transdermal drug delivery system. It was shown that the proposed multi-layer patch, including a microfiber heater and drug eluted hydrogel, could be used as a wearable wound care system, suitable for gastric and hepatic injuries [84].

### 5.2. Light–Responsive Drug Release

Remote, stable, and instant delivery, in addition to temporally and spatially controlled release behavior, are among the significant advantages of light-responsive hydrogels, compared to other types of stimuli-responsive hydrogels [85,86]. Photodegradable hydrogels, which are widely utilized as light-responsive hydrogels, function in response to UV or NIR light. They are triggered using light sources, and are capable of releasing their cargo in a controlled manner. The energy absorbed from light isomerizes the polymer and eventually leads to degradation [87,88]. Using this platform, light-responsive hydrogel microbeads composed of poly (N-isopropylacrylamide-co-vinyl-2-pyrrolidinone) were used for transdermal on-demand drug delivery [89]. According to Figure 4E, it was demonstrated that light triggering of magnetic nanoparticles incorporated hydrogel could indirectly generate heat and increase the temperature of the matrix, resulting in bead shrinkage. This behavior was successfully used for the on-demand drug release, which was proportional to the intensity of the light source.

Multifunctional polymer nano-gels have soft physical properties, resembling natural extracellular matrices, and possess a high loading capacity, owing to their large surface area and porous structure [90]. Combining this capability of polymer nano-gels and the unique photothermal conversion ability of fluorescent carbon nanoparticles (FCNPs) has been widely utilized for developing multifunctional applications in DDS [91]. For instance, Wang et al. [92] studied a multifunctional PNIPAAm–FCNP hybrid nano-gel and developed a model that possesses high drug release controllability through exogenous irradiation with near-infrared (NIR) light. NIR-responsive supramolecular hydrogel, as an example of on-demand degradable hydrogel, was developed by encapsulating nanoparticles in poly (ethylene glycol) (PEG) and alpha-cyclodextrin (α-CD). It was observed that the hydrogel degradation from NIR exposure could locally deliver drugs, which could have applications in chemotherapy. NIR light source was also used by Anugraha et al. to fabricate an NIR-responsive alginate-based hydrogels using diselenide containing cross-linkers and NIR sensitive indocyanine green (ICG) as a remote-controlled doxorubicin (DOX) carrier [93]. Under NIR-light, ROS generated by the ICG decomposed the diselenide bonds in the hydrogel matrix, resulting in the release of entrapped DOX [93].

As another example of light-responsive polymers, a photo-responsive monomer was designed, and the related poly(S-(o-nitrobenzyl)-L-cysteine)-b-poly(ethylene glycol) (PNBC-b-PEO) block copolymers were synthesized to achieve light-triggered drug release [94]. Nanoparticles were loaded with doxorubicin to induce controlled-release, by gradually photocleaving of their poly(S-(o-nitrobenzyl)-L-cysteine)-b-poly (ethylene glycol) (PNBC) core. Sustained drug release after UV irradiation at 365 nm for different times showed that this fabrication strategy has the potential for anticancer therapy.

### 5.3. Ultrasound–Responsive Drug Release

Ultrasound-responsive drug release is a new class of noninvasive and cost-effective stimulated DDS, which functions with the simulation of ultrasound [95,96,97]. Ultrasound is among the physical therapy modalities with high spatial and temporal controllability necessary for on-demand drug delivery. In ultrasound-responsive chemotherapeutic systems, the injected drug-loaded nanodroplets are converted into microbubbles, under the influence of ultrasound energy [98,99,100].

As an example, perfluorohexane nanodroplets with the shell of chitosan were synthesized through emulsion chemistry, and were subsequently loaded with curcumin [101]. The loaded nanodroplets were successfully able to have on-demand release the drug in response to external ultrasound stimuli. In vitro cytotoxicity of curcumin-loaded nanodroplets was studied on 4T1 human breast cancer cells, and showed an enhanced inhibitory effect on the growth of cancer cells.

Another model of nano-sized DDS was developed by Uesugi et al. [102], which had a tissue-type plasminogen activator (t-PA) with the ability to recover under ultrasound exposure. Different amounts of ethylenediamine were added to cationized gelatins, which was then surface-modified by grafting polyethylene glycol (PEG). They also studied the cytotoxicity of their modified hydrogel and body distribution of t-PA, which indicated that the developed nano-DDS could serve as an effective therapeutic platform.

## 6. Endogenously Triggered Drug Release

Using the body’s specific microenvironments as an internal stimulus is one way of endogenously triggering drug release [103]. pH-responsive carriers were designed as a smart DDs, in order to obtain greater control over drug release in response to a specific physiological stimulant. Greater control would simultaneously enhance treatment outcomes and minimizing side effects. Conformational changes in the ionization state of polymers prompt chemical reorientations of functional groups, which affects polymer-drug interactions. In addition, the incorporation of various reduction and oxidation responsive side chains to the polymer improves the smart drug carrier’s efficacy in on-demand drug delivery applications. In the meantime, designing drug carriers with specific structures and dual responsivity has gained considerable attention; however, lowering the cost is still a challenge [104].

### 6.1. Redox-Responsive Controlled Release

**Figure 5:**
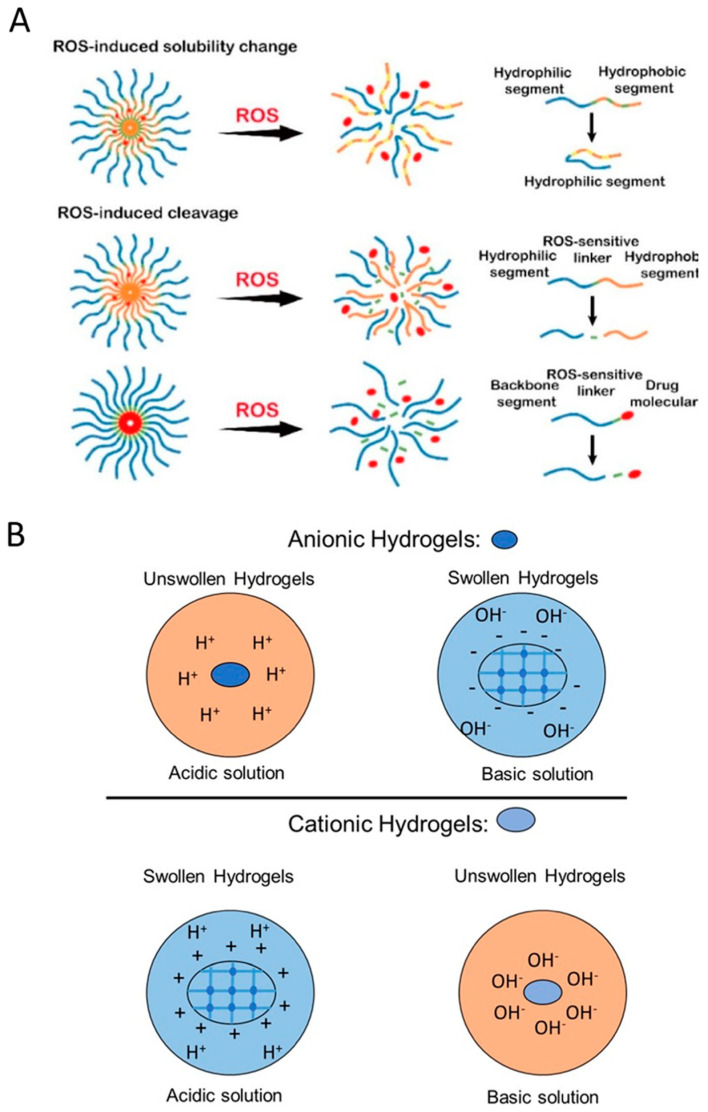
(**A**) Schematic of reactive oxygen species (ROS)-sensitive drug delivery mechanism including ROS-mediated solubility change and ROS-mediated cleavage of hydrogels. Adapted with permission from Tu et al. [108]. (**B**) Schematic of pH-responsive swelling of anionic and cationic hydrogels in acidic and basic solutions.

The main principle of the redox-responsive polymeric DDs is based on the differences in reduction potential that exist between tumors and healthy tissues [105]. The glutathione (GSH)/glutathione disulfide (GSSG) has been known to be the most plentiful redox couple in animal cells. In vivo research in mice has shown that the GSH in tumors is at least 4-fold higher than their counterparts in healthy cells [106]. The differing concentrations of GSH found in extracellular and intracellular fluid in response to biological reducing conditions, such as tumor sites, activate the specific anti-tumor drugs [107,108]. Designing redox-responsive polymers and their conjugates usually involves the incorporation of redox-sensitive components, such as disulfide linkage in the main chain, the side chain, and through the cross-linker, and exploiting oxidative microenvironments in the body as triggers for therapeutic release [108]. As shown in Figure 5A, the solubility of the ROS-sensitive polymers changes upon exposure to ROS-rich environment results in cleavage, swelling, and degradation of the drug carriers.

#### 6.1.1. Systems with the Disulfide Linkage

Rapid cleavage of disulfide bonds by GSH can be used to attain reduction-responsive polymeric DDs [107]. Thiol-disulfide exchange reactions, which have been widely used for crosslinking structures, such as shell cross-linked micelles, interlayer-cross-linked micelles, and gels, are an effective method for inducing disulfide linkage. Dithiodipropionic acid, bis (2,2′-hydroxyethyl) disulfide, cysteamine, and their derivatives are the most useful crosslinking agents with redox sensitivity [108]. In addition to the development of the disulfide-containing cross-linkers, atom transfer radical polymerization, reversible addition-fragmentation chain transfer polymerization, and ring-opening polymerization have been recently employed for introducing disulfides as end-groups. Adding disulfides to olefins results in the development of polymers with redox-sensitive side chains. This technique maintains a balance between the hydrophobic and hydrophilic blocks of copolymers and the breakage of this balance by cleaving the disulfide bond results in the drug release [104]. As an example, disulfide-containing poly (amido amine) is grafted with siRNA to be used as a potential local gene delivery carrier in tumor area [107].

#### 6.1.2. Systems with the Diselenide Linkage

Selenium and sulfur have many similar chemical properties. These elements can be utilized for the same purpose of developing redox resistive drug carriers [109]. Polymers containing diselenide were found to be more responsive in a reducing environment, due to their lower bond energies in comparison with their sulfide counterparts [109,110]. Selenium-containing molecules introduce a strong antitumor tendency by generating ROS and inducing apoptosis of tumor cells. However, incorporation of the diselenide bonds is difficult, and the compound’s use is limited by the insolubility of selenide in aqueous solutions [111].

### 6.2. Oxidation-Responsive Drug Release

Oxidation-responsive drug carriers mainly target ROS, such as hydrogen peroxide (H_2_O_2_) and hydroxyl radicals [109]. Like glutathione, ROSs are involved in some serious diseases, including arteriosclerosis, heart defects, and cancer. The pathological signals employed to obtain redox sensitivity are produced by any disorder that results in cell damage. Sulfur-based materials, boronic ester groups, and phenylboronic acid derivatives can be used as oxidation-sensitive substitutes in DDs, to improve the properties of redox-responsive drug carriers. In addition, monitoring localized ROS levels improves the diagnosis and the treatment of a wide range of diseases, including cardiovascular diseases and drug-induced organ failure. Incorporating ROS-responsive materials were also explored for activating drug release in inflammatory tissues [107] Oxidation-responsive carriers include polymers with oxidation-sensitive chains such as thioether, selenide/telluride, diselenide, thioketal, aryl boronic ester, peroxalate ester, boronic ester, and polyproline [103,104].

#### 6.2.1. Systems with the Disulfide Linkage

Poly (propylene sulfide) was the first hydrophobic block introduced for oxidation-responsive drug delivery. Exposing H_2_O_2_ to disulfide-containing polymers results in the oxidation of the sulfide groups into hydrophilic sulfoxide and ultimately into sulfone [107]. Sulfide-containing oxidation-sensitive polymers generally have a linear and stable structure that is not amenable for drug delivery applications [104]. Various forms of disulfide components have been designed to expand oxidation sensitive vehicles for biomedical applications, including micelles, nanoparticles, microspheres, vesicles, fibrils, and hydrogels [109].

#### 6.2.2. Systems with Thioether Linkage

Thioether-containing polymers are the most popular ROS-responsive material utilized in biomedical applications. Thioether-containing polymers experience a phase transition from a hydrophobic (sulfide) state to a hydrophilic (sulfoxide-sulfone) state in the presence of an oxidative microenvironment. This phase transformation leads to the destabilization of the carrier, resulting in drug release [112]. Incorporation of a ketal into the poly-thioether prompts the development of dual stimuli-responsive polymers for protein delivery. While ketal groups provide pH-sensitive degradation properties, diffusion of H_2_O_2_ oxidizes sulfide groups leads to the hydrophobicity of the carrier, causing drug release [103].

#### 6.2.3. Systems with Selenium-Telluride Linkage

Selenium and Tellurium-based compounds are well-known to have higher oxidation and reduction-sensitivity, in comparison to sulfides from the same group on the periodic table. These compounds are used for drug delivery applications, and their release mechanism is similar to the oxidation of sulfide groups. Selenium and telluride are initially water-insoluble and hydrophobic, and become hydrophilic and water-soluble in ROS-rich environments [104]. Tellurium-containing polymers are thought to be more oxidation-responsive, due to their lower electronegativity, and have lower toxicity in comparison with selenium-containing polymers. While selenium-based polymers could provide excellent antitumor efficiency, their final residues can be removed by enzyme digestion after therapeutic release [103].

#### 6.2.4. Systems with Ferrocene Linkage

Ferrocene-containing polymers have unique oxidation and redox properties, which makes them amenable for designing drug carriers with oxidation-sensitivity in a wide variety of applications, such as in biomedicine, as biosensors, actuators, batteries, and liquid crystals. The hydrophobic state of the ferrocene unit can be changed to the hydrophilic state during charged ferrocenium cation generation in response to the oxidation process [103]. Based on the electrical conductivity of ferrocene-containing polymers, they can serve as dual-responsive drug carriers, which are suitable for long-term drug release for achieving maximal therapeutic effects [104].

#### 6.2.5. Systems with Boronic Esters

Boronic esters are commonly exploited for oxidation-induced degradation, and they can be utilized as triggers for controlled burst release. Boronic ester-containing polymers are generally conjugated to imaging agents and anti-cancer drugs. These agents and drugs are then released upon exposure to stimuli [103,104]. For example, after the incorporation of boronic ester, the water-soluble polysaccharide becomes organically soluble, improving the encapsulation process. After oxidation of the boronic esters, the polysaccharides are degraded entirely into small molecules and release the drug [103]. Hydroxyl-modified dextran with aryl boronic ester follows the same mechanism for drug release [93,103].

### 6.3. pH-Responsive Drug Release

pH variations have been used to control the delivery of therapeutic agents in specific organs, such as the gastrointestinal tract, or intracellular compartments [113]. They are also used to activate drug release, in response to microenvironmental changes resulting from pathological situations, such as cancer or inflammation. Developing hydrogel formulations that are responsive to environmental stimuli provides the opportunity for dose and target specific therapeutics [107,114]. By extending the knowledge of pH-responsive mechanisms, a hydrogel can be designed to respond to microenvironmental stimuli and trigger physical and chemical reactions, such as shrinking, swelling, membrane fusion, and membrane disruption at a specific pH [115,116]. The pH responsivity of hydrogels is attained through the protonation and deprotonation of weak poly-basic, or poly-acidic copolymers, or the degradation of acid-cleavable bonds in various pH conditions [105].

#### 6.3.1. Mechanism of Action of pH-Responsive Targeted Delivery

Polymers with weak acidic/basic functional groups have been used as pH-sensitive polymers. These groups are prone to accepting protons at acidic pHs and releasing them at basic pHs. Electrostatic interactions and charge transitions result in generating polyelectrolytes depending on their p*K*a values. The electrolytes have a unique ability to tune their solubility through hydrophobic/hydrophilic transitions causing swelling/deswelling, which, in turn, results in drug release upon exposure to different pHs [117]. The ionized polymers with 3–10 p*K*as are considered to have the potential for pH-responsive drug delivery applications. The electrostatic interactions and deionization of the functional groups in acidic or basic environments lead to conformational changes, depending on the ionization state of the polymers. pH-induced ionization/protonation of the functional groups, such as amino, imidazolyl, sulfonates, and carboxyl groups, results in the hydrophilic-hydrophobic phase transition of the polymers [118]. Protonation is known to be the most commonly used technique for designing pH-triggered drug carriers. Based on the ionization mechanism and the functional groups, polymers are divided into anionic/cationic polyelectrolytes and neutral polyampholytes, depending on their charge status at neutral pH. Figure 5B illustrates the schematic of swelling for both anionic and cationic polyelectrolyte in acidic and basic environments.

#### 6.3.2. Anionic pH-Responsive Hydrogels

##### Hydrogels with Carboxylic Groups

Anionic polymers with carboxylic functional groups are utilized for targeting acidic pH microenvironments. The carboxylic groups are deprotonated and hydrophilic at a neutral pH of 7.4, forming anionic polyelectrolytes, and become protonated and hydrophobic in acidic conditions. The molecular rearrangement is a function of electrostatic interactions, resulting in the deformation of the carrier, which causes drug release. Anionic groups with carboxylic functional groups, including Poly (aspartic acid) [119], Poly (acrylic acid) [120], Poly (ethyl acrylic acid) [121], Poly (N-isopropyl acrylamide-co-acrylic acid) [67], Poly (methacrylic acid) [122], and 3-methylglutarylated poly (glycidol) [123], have various p*K*a values between 4.80–6.50, and release therapeutic agents in acidic conditions beneath their p*K*a. The other functional group that plays a significant role in drug release from anionic groups is the sulfonamide group. Polymers with poly-sulfonamides groups have a wide range of p*K*as, from 3 to 11, based on the substituents of their chemical structure. However, sulfonamides with p*K*a values of 7.4 are the most promising polymers.

##### Hydrogels with Sulfonic/Phosphonic Acid Groups

Anionic polymers with sulfonic/phosphonic acid functional groups are generally known for being hydrophilic in their anionic state at acidic pHs and for swelling above their p*K*a value. The polymers with sulfonic acid groups/side-chains include poly (2-acrylamide-2-methylpropane sulfonic acid), poly (4-styrene sulfonic acid), and functionalized styrene−divinylbenzene copolymers, are examples of anionic polymers with phosphonic acid groups [124,125].

##### Hydrogels with Boronic Acid Groups

Boronic acid-containing polymers are generally utilized as self-healing gels or glucose sensors with unique electronic and physicochemical properties. In contrast to the low molecular weight of boronic acids, the incorporation of this compound into polymers results in swelling, but not dissolution in the aqueous solutions [126]. Phenylboronic acid moieties-containing polymers are commonly developed using a variety of techniques, such as an additional deprotection process [117].

#### 6.3.3. Cationic pH-Responsive Hydrogels

This group of polymers generally includes ionizable tertiary amine groups in their side chains. Amine groups accept protons at acidic pH and donate protons at basic pH, and can be utilized as triggers for the therapeutic release of drugs. Poly (β-amino ester) [127], Poly (N,N-dimethylamino) ethyl methacrylate [128], and poly (l-histidine) [129] are some examples of cationic polymers with a p*K*a value of 6.5-7. The incorporation of other functional groups, such as Poly (vinyl pyridine), imidazole, piperazine, pyrrolidine, and morpholino, leads to a wide variety of cationic polymers [117]. Cationic polymers have a greater toxicity than anionic polymers. However, the negative charge of anionic polymers results in lower uptake levels by cells, due to charge repulsion. A combination of polymers with both a shielding and a de-shielding effect was introduced to address this problem. The cationic polymer was covered with an anionic pH-responsive polymer to ameliorate the negative effects and to improve cell adhesion [116].

#### 6.3.4. Neutral pH-Responsive Hydrogels

Except for the anionic and cationic polyelectrolytes, neutral polyampholytes have been widely utilized in biomedical applications, due to their non-cytotoxicity and biodegradable nature. Natural polymers, such as dextran, hyaluronic acid, alginic acid, chitosan, and gelatin, have been exploited to develop pH-sensitive carriers at two p*K*a values, and used for developing pH-responsive, self-healable, and tissue adhesive polymers [117,130].

## 7. Bio-Responsively Triggered Drug Release

### 7.1. Inflammatory-Responsive Drug Release

Inflammatory tissue responses generally appear at injury sites. Inflammation-responsive cells, such as macrophages and polymorphonuclear leukocytes (PMNs), play a key role in the normal healing processes after injury. One of the major products of inflammation-responsive cells at the injured sites and tissues are hydroxyl radicals (OH^-^), derivatives of oxygen metabolites. Utilizing these products to design bio-responsive hydrogels is a promising strategy in the local delivery of different anti-inflammatory agents.

In this way, Yui et al. introduced a hydroxyl radical-responsive DDs [131]. They utilized hyaluronic acid (HA), a linear mucopolysaccharide consisting of repeating units of N-acetyl-d-glucosamine and d-glucuronic acid, for preparing inflammation-responsive hydrogels. Due to the destructiveness of hydroxyl radicals, they can effectively degrade HA. HA degradation in response to hydroxyl radicals was detected only at the surface of the hydrogel, indicating surface erosion degradation, which resulted in the release of anti-inflammatory drugs. Further utilization of these hydrogels involved the introduction of microspheres as a drug carrier inside the hydrogels’ structure, through which the sustained release of drugs from the microspheres follows the surface erosion of the hydrogels. These HA gels are widely used in vivo researches as inflammation-induced DDs, in particular for chronic inflammatory problems, such as rheumatoid arthritis [132].

Myocardial infarction (MI), resulting from the blockage of the coronary arteries, is a leading cause of death worldwide. An MI, leads to the overproduction and release of large amounts of reactive oxygen species (ROS) in the microenvironment. This leads to disruptions in cellular homeostasis, which damage cardiomyocytes, stimulates inflammation, and induces fibrosis. The majority of cardiac patches are derived from common biomaterials that induce up to a 3-fold release of ROS at the surgery site. Accordingly, ROS-responsive biomaterials are ideal for the delivery of anti-inflammatory drugs following an MI. In addition to ROS-triggered drug release, polymers with ROS-responsive bonds in their backbones or side chains are capable of consuming the excessive ROS produced by the MI. This reduces injury to the cardiac tissue, and has the potential for preventing and treating cardiovascular disease [133,134,135]. Yao et al. reported a biodegradable elastomeric polyurethanes (PUTK) with ROS-responsive properties, which they made fibrous patches from. The patches were subsequently loaded with methylprednisolone (MP) and used in the in vivo treatment of post-MI rats [136]. Hydrogel scaffolds degraded by ROS are excellent candidates for tissue regeneration. Poly(thioketal) (PTK) urethane (PTK-UR) is one of the most studied ROS-degradable scaffolds (based on ROS-cleavable PTK macrodiols), and has been used to promote diabetic wound healing. The thioketal group has been used to design orally-delivered nanoparticles. These nanoparticles remain stable in the stomach, and release their cargos at the site of ulcerative colitis. The thioketal linkages are cleaved by H_2_O_2_, cleaving the polymer chain and releasing the drug [137].

### 7.2. Enzyme-Triggered Drug Release

Enzyme responsive materials have the great potential for the design of intelligent DDs. Some enzymes are overexpressed in the diseased area, and many of these enzymes possess the ability to catalyze the cleavage of chemical bonds, such as ester bonds and amido bonds, which can be used as triggers for drug release. Woo et al. prepared a polymer prodrug by connecting a polymer chain to a ciprofloxacin via ester groups and investigated the effect of esterase on the drug’s release characteristics [138]. The local inflammatory environment, induced by the implant-associated infection, was a critical factor in generating of HO^-^ [139]. Local inflammation usually leads to esterase overexpression, which could be used as a trigger in the design of inflammation-responsive drug release systems, by utilizing esterase’s catalytic activity on ester groups [140]. Zhang et al. made inflammation-targeting hydrogel microfibers that were loaded with anti-inflammatory agents [141]. The microfibers showed preferential adhesion to the inflamed epithelial surface, and the drug was released with the rupture of hydrogel capsule in inflamed tissue, under the catalysis of the overexpressed esterase [142].

## 8. Potential Clinical Usage of Stimuli Responsive Hydrogels

### 8.1. Wound Healing

**Figure 6:**
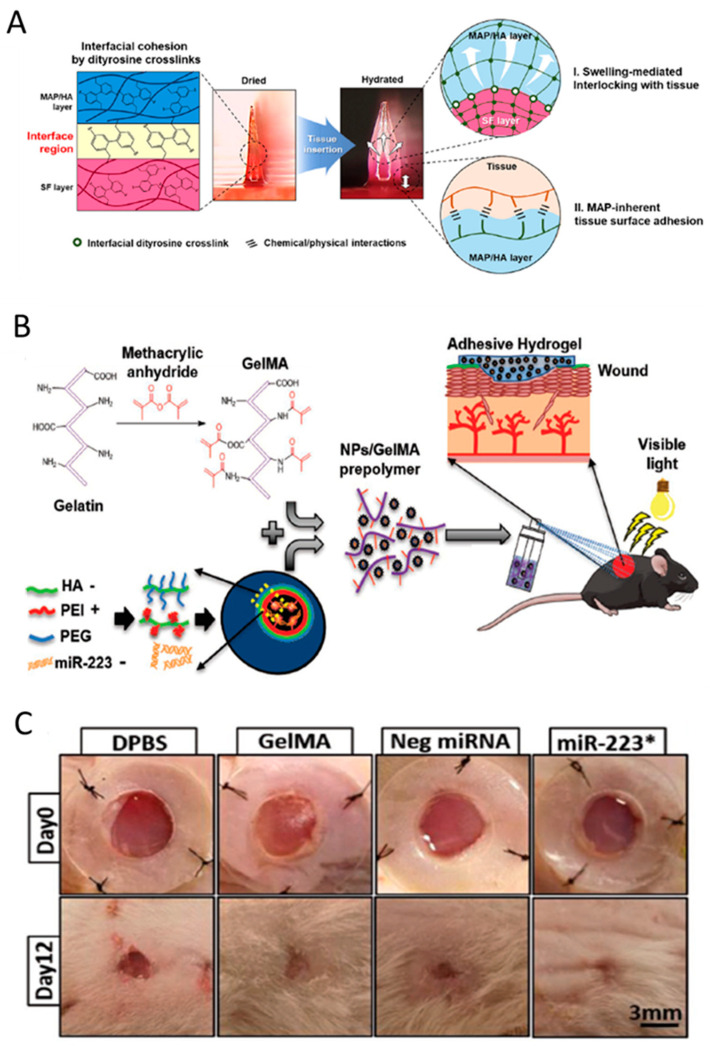
(**A**) Illustration of the mechanisms of a hydrogel-forming adhesive microneedle patch consisting of a mussel adhesive protein-based swellable and sticky shell and a silk fibroin-based non-swellable core. It shows the structure and the reaction of the fabricated patches at the interface with the tissue. Adapted with permission from Jeon et al. [143]. (**B**) Synthesis illustration of the process for the formation of hyaluronic acid nanoparticle/miR-223*-laden GelMA hydrogels and the use of these adhesive hydrogels for wound healing. Adapted with permission from Gao et al. [144]. (**C**) Images of the wounds treated with Dulbecco’s phosphate-buffered saline (DPBS), GelMA, Neg miRNA incorporated GelMA, and miR-223* incorporated GelMAat days 0 and 12 of treatment. * Images for animals at day 12 were acquired after removal of the silicon splint to highlight the extent of wound healing. Adapted with permission from Saleh et al. [144].

One of the most usable clinical fields of the smart hydrogels is wound healing. According to the literature an ideal and functional tissue adhesive for wound closure and treatment is: (i) biodegradable with no toxicity (ii) rapidly cross-linkable and easily applicable, (iii) antimicrobial and impervious to antibiotic resistance, (iv) strongly adhesive, (v) tunable and long-lasting, and (vi) a promotor of tissue regeneration and wound healing. Therefore, a new hydrogel-based biomaterial is needed to be used as a carrier for local antimicrobial drugs, as well as a regenerative growth factor carrier. Effective and prompt wound closure is crucial to recovery and preventing complications. Jeon et al. reported an implantable adhesive microneedles (MN) patch, comprised of a swellable mussel adhesive protein (MAP)-based shell and a core of non-swellable silk fibroin (SF) [143]. As shown in Figure 6A, the MN patch demonstrated superior wound healing properties against luminal leaks.

As another example, Saleh and his coworkers reported a sprayable and adhesive form of GelMA hydrogel for the delivery of miRNAs to non-healing wounds. They used miR-223, a potent regulator of bone marrow-derived cells, to load the HA nanoparticles (NPs), and delivered it by a sprayable hydrogel. GelMA/NP/miR-223* hydrogels could drive wound healing by not only triggering the resolution of the inflammatory phase but also promoting the formation of new vascularized skin tissue (Figure 6B,C) [144].

### 8.2. Spinal Cord Injury Treatment

**Figure 7:**
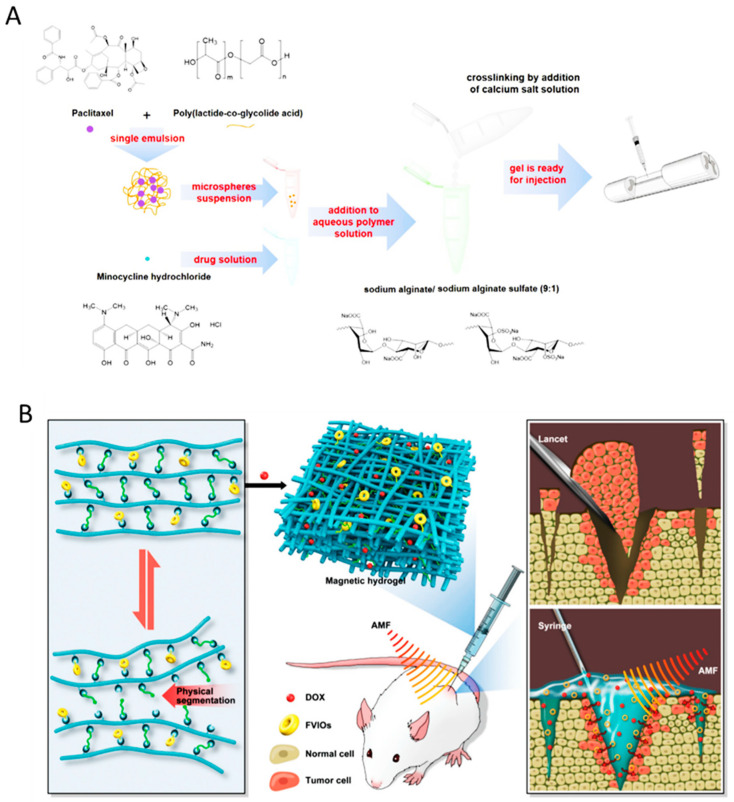
(**A**) Spinal cord injury treatment using alginate hydrogel preparation encapsulated with multi-drug loaded PLGA particles for injection into the lesion site. Adapted with permission from Nazemi et al. [145]. (**B**) Localized administration of vortex-domain iron oxide-functionalized magnetic hydrogel for breast cancer postoperative recurrence prevention using its pH-responsivity. Adapted with permission from Gao et al. [148].

Spinal cord injury (SCI) is a debilitating neurological condition caused by mechanical trauma to the spinal cord. Generally, spinal cord injury includes a series of events, such as vascular changes, free radical formation, the disruption of ionic balance, apoptosis, and inflammatory responses are triggered by spinal cord injury. Considering post-surgery local treatment of spinal injury, Nazemi et al. administrated, a combination of neuroprotective drug, minocycline hydrochloride (MH) and neurodegenerative drug, paclitaxel (PTX) into the lesion site of a hemi-section model of spinal cord injury in rats at acute phase [145]. An affinity injectable alginate hydrogel, based on electrostatic interactions, and metal-ion chelation that incorporated MH, a water-soluble small molecule drug, was developed for this purpose. Subsequently, PTX was encapsulated in the PLGA microspheres and inserted in the alginate hydrogel (Figure 7A). They demonstrated that the dual-drug treatment was capable of reducing inflammation, enhancing neurogenesis, and promoting functional recovery [145].

### 8.3. Cancer Treatment

Due to the complexity of tumor microenvironment (TME) and micro-physiological barriers of TME, which impede the efficient systemic drug delivery, various types of drug-loaded hydrogels have been introduced for administrating local chemotherapeutics to tumor sites.

Considering the significance of efficient local drug delivery to tumor burden, GlioMesh was developed using 3D printing of an alginate-based hydrogel loaded with temozolomide (TMZ)-releasing PLGA microparticles, which is capable of delivering TMZ over several weeks at the brain tumor site. It was shown that the level of autophagic activity and a larger degree of mitochondrial induced damage, as an indication of drug toxicity, was significantly higher in using GlioMesh in comparison with free TMZ administration. Delivering immunotherapeutic drugs with a lower systemic toxicity and side effects is a predicament that is addressed using engineered hydrogels. To meet this goal, Duong et al. utilized a degradation-adjusted injectable smart hydrogel to enhance the humoral immune response and potentiate antitumor activity in human lung carcinoma. Poly(esters) and levodopa (DOPA) have been used as thermo-responsive and non-toxic hydrogels and incorporated into the HA matrix. This smart biodegradable hydrogel enhanced the recruitment of DCs into the hydrogel structure and activated immune cells against the specific tumor antigens. This hydrogel, as a successful cancer vaccine, generated a strong antigen-specific humoral response, and provided prophylactic efficacy against a murine B16/OVA melanoma [146]. In another study, Zhang et al. fabricated fibrin hydrogel to delivery of CTX and anti-PDL1 for localized chemo-immunotherapy after the resection of primary tumors [147]. Maximized synergistic anticancer efficacy was obtained by different release kinetic of CTX and anti-PDL1 [147].

Engineering the chemical composition and structure of the hydrogels is another promising method to generate suitable hydrogel carriers for local treatment of the tumor. Functional micro and nanoparticles are considered as additives used for modifying the chemical structure and properties of the hydrogels used for local drug delivery and cancer recurrence prevention. A magnetic hydrogel comprising ferromagnetic vortex-domain iron oxide (FVIOs) dispersed in Glycol chitosan was reported by Gao et al. [148]. (Figure 7B). This nanocomposite hydrogel exhibited promising properties, such as self-healing, self-conformal ability, controlled pH-sensitive drug release, and biodegradability. Compared to chemotherapy and hyperthermia, the magnetic hydrogel showed higher efficacy in local tumor recurrences suppression [148]. Locoregional chemotherapy, directly delivering anti-tumor drugs at the tumor site, is feasible using forms of implantable, injectable, and sprayable hydrogels. In this regard, Wu and his coworkers reported a multi-functional injectable hydrogel for the locoregional delivery of chemotherapeutic drugs with high efficacy. Phenylboronic acid-modified mesoporous silica nanoparticles (PBA-MSNs) and dopamine-conjugated hyaluronic acid (DOP-HA) hydrogel were significant components of the injectable hydrogel. Acid-cleavable dynamic boronate bonds were produced by the interaction of DOP-HA with PBA groups, and Doxorubicin controlled release was obtained in response to TME pH condition, after NPs successfully penetrated the tumor cell [149].

### 8.4. Periodontal Regeneration

Periodontitis is a disease caused by complex interactions between the host immune system and plaque microorganism that leads to inflammation. Periodontitis can induce alveolar bone resorption, which is one of the main causes of tooth loss in adults. To terminate alveolar bone resorption, simultaneous anti-inflammation and periodontium regeneration are required to address this challenge, making user-friendly forms of hydrogels suitable for this application. Considering the requirement of local drug delivery to periodontal tissue, Xu et al. reported an injectable and thermo-responsive hydrogel consisting of chitosan, β-sodium glycerophosphate, and gelatin for the continuous delivery of aspirin and erythropoietin. This delivery system was quite effective in anti-inflammation and periodontium regeneration [150]. In other research, Dong et al. reported a thermoresponsive injectable PVA based hydrogel to control the release rate of antibiotics [151]. The sustained release of the drug endowed a prolonged bacteriostatic ability, while Chitosan decorated metronidazole (MTZ) enhanced the injectability and bio-adhesive property of the hydrogel [151].

## 9. Conclusions and Outlook

Surgery is the primary step in the treatment of many diseases, such as cancer, periodontal, bone-related, and wound-based diseases. However, in several cases, surgery alone cannot be considered the only treatment modality, due to its inability to eliminate the disease completely, its invasiveness, and serious side effects such as pain, inflammation, and infection. As such, surgery is generally followed by other treatment modalities, such as radio- and chemotherapy, to achieve the best clinical outcomes. Localized delivery of therapeutics has recently become an effective method for the post-surgical management of many diseases, as it enables the delivery of higher doses of drugs to the disease site with reduced systemic toxicity to vital organs, such as the liver, kidneys, and the heart. Owing to their desired cytocompatibility, tunable physicochemical properties, controllable degradability, and ability to protect labile drugs from degradation, hydrogels have become attractive biomaterials as drug depots for localized delivery of therapeutics. Hydrogels for drug delivery are applied in three modes—implantation, injection, or by spraying. The selection of each method depends on the hydrogel’s physical properties, ease of implementation, need for delivering drugs topically or deep into tissues, and the mode of drug delivery (i.e., burst, sustained, or controlled release). Implantable hydrogels with strong mechanical properties that possess adjustable physical properties for on-demand drug release are the best candidate for local delivery. Furthermore, in specific cases like trauma-based diseases, the regeneration capability of such hydrogels is prominent. Additionally, loading growth factors or stem cells into the implantable hydrogel structure is a great opportunity for accelerating the regeneration process in the injured tissue. However, most implantable hydrogels require invasive surgeries to insert the material at the site of the disease. As such, follow-up surgeries may be required to replace the implant, if frequent drug delivery is desired. Injectable hydrogels, on the other hand, can be delivered via needles or medical catheters, allowing minimally-invasive deployment of the drug depot. Therefore, injectable hydrogels are attractive materials for delivering drug during chemotherapy, where several cycles are needed to complete the treatment. Sprayable hydrogels, have also been widely used for delivering drugs to large surfaces during surgical operations or after the surgery is completed. The ease of deployment and ability to cover large areas rapidly has made sprayable hydrogels as attractive drug-delivery materials for management of large burn wounds and bone defects.

The ability to change the release rate of drugs or deliver different drugs at each stage of the disease is highly desirable. In that regard, intelligent hydrogels that undergo three-dimensional structural changes in response to an environmental stimulus hold great promise to deliver drugs “when” and “where” needed. With recent advancements in material science, hydrogels depots can be engineered that specifically release their payloads in response to the presence of endogenous or exogenous triggers. Such a unique feature is beneficial for treating cancers or infected wounds by preventing chemo or antibacterial resistance, and improving the clinical outcomes of the treatment strategy. Having a facile preparation process and intra-tumor injectability, as well as the ability for smart drug release, makes the injectable and sprayable hydrogels potential candidates for cancer treatment strategies. However, despite extensive research on various types of injectable hydrogels in cancer treatment, periodontal, and wound healing in preclinical settings, challenges regarding the translation of DDSs to clinical use, including human safety, multi-organ side effects, large scale manufacturing, and cost-effectiveness in comparison to current therapies, still remain. Therefore, additional studies are required to understand the critical tenets of local-based DDSs that are specific to each patient. For a better clinical target and tuning the release profile in accordance to each patient’s condition, the structural modification of hydrogels, such as using novel nano and microparticles as cargo in the hydrogel’s matrix, is essential. These modifications aim to adjust the initial burst release of drugs and endow multi-responsive properties to DDSs. Moreover, hydrogels should be able to provide a platform for the localized combination of chemo and immune therapies. Additionally, the in-depth characterization of the drug release kinetics of therapeutics, mathematical modeling, and simulation is essential for reducing the cost of DDSs.

## Figures and Tables

**Table 1:** Injectable hydrogel systems and their applications.

HYDROGEL	APPLICATIONS	REF.
CHITOSAN/PEGDA	Delivery of DOX for HCC cancer therapy	[53]
CHITOSAN/DABC	Injectable wound dressing with antibacterial properties	[68]
HYALURONIC ACID	Delivery of doxycycline and doxorubicin	[55]
ALGINATE/Hap/GMs	Delivery of tetracycline hydrochloride and bone tissue engineering	[57]
ALGINATE/PNIPAAm	Delivery of DOX for multidrug resistance cancer cells	[56]
COLLAGEN/GNPs	Delivery of TMPyP (a photosensitive drug) for cancer therapy	[58]
GELATIN/OSA/ADH	Delivery of Human epidermal growth factor and encapsulation of cells	[59]
CHONDROITIN SULFATE/PVAMA	Delivery of cationic therapeutics in oncology therapeutics	[60]
PKP	Delivery of paclitaxel for cancer chemotherapy	[61]
PEG	Delivery of growth factors for vascularization	[62]
PLGA	intra-bone delivery of sodium alendronate (Aln)	[63]
PEG/SILK	Delivery of micronized dexamethasone (mDEX)	[64]
PLGA/PEG/PLGA (triblock copolymer)	Delivery system for tissue engineering and eye/ear diseases	[65]
PEG/PCL	Delivery of paclitaxel	[66]
